# Nucleoside analogs NM107 and AT-527 are antiviral against rubella virus

**DOI:** 10.1093/pnasnexus/pgad256

**Published:** 2023-08-03

**Authors:** Mark Dittmar, Kanupriya Whig, Jesse Miller, Brinda Kamalia, Suganthi Suppiah, Ludmila Perelygina, Kathleen E Sullivan, David C Schultz, Sara Cherry

**Affiliations:** Department of Pathology and Laboratory Medicine, University of Pennsylvania, 3450 Hamilton Walk Philadelphia, PA 19104, USA; Department of Microbiology, University of Pennsylvania, 3450 Hamilton Walk Philadelphia, PA 19104, USA; Department of Biochemistry and Biophysics, University of Pennsylvania, 3620 Hamilton Walk Philadelphia, PA 19104, USA; Department of Pathology and Laboratory Medicine, University of Pennsylvania, 3450 Hamilton Walk Philadelphia, PA 19104, USA; Department of Biochemistry and Biophysics, University of Pennsylvania, 3620 Hamilton Walk Philadelphia, PA 19104, USA; Division of Viral Diseases, Centers for Disease Control and Prevention, 1600 Clifton Rd. Atlanta, GA 30329, USA; Division of Viral Diseases, Centers for Disease Control and Prevention, 1600 Clifton Rd. Atlanta, GA 30329, USA; Division of Allergy and Immunology, Children's Hospital of Philadelphia, 3615 Civic Center Blvd., Philadelphia, PA 19104, USA; Department of Biochemistry and Biophysics, University of Pennsylvania, 3620 Hamilton Walk Philadelphia, PA 19104, USA; Department of Pathology and Laboratory Medicine, University of Pennsylvania, 3450 Hamilton Walk Philadelphia, PA 19104, USA; Department of Microbiology, University of Pennsylvania, 3450 Hamilton Walk Philadelphia, PA 19104, USA; Department of Biochemistry and Biophysics, University of Pennsylvania, 3620 Hamilton Walk Philadelphia, PA 19104, USA

**Keywords:** rubella, antivirals, high-throughput screening, nucleoside analogs

## Abstract

Rubella is a highly contagious viral infection that usually causes a mild disease in children and adults. However, infection during pregnancy can result in a fetal or newborn death or congenital rubella syndrome (CRS), a constellation of permanent birth defects including cataracts, heart defects, and sensorineural deafness. The live-attenuated rubella vaccine has been highly effective, with the Americas declared free of endemic rubella transmission in 2015. However, rubella remains a significant problem worldwide and the leading cause of vaccine-preventable birth defects globally. Thus, elimination of rubella and CRS is a goal of the World Health Organization. No specific therapeutics are approved for the rubella virus. Therefore, we set out to identify whether existing small molecules may be repurposed for use against rubella virus infection. Thus, we performed a high-throughput screen for small molecules active against rubella virus in human respiratory cells and identified two nucleoside analogs, NM107 and AT-527, with potent antiviral activity. Furthermore, we found that combining these nucleoside analogs with inhibitors of host nucleoside biosynthesis had synergistic antiviral activity. These studies open the door to new potential approaches to treat rubella infections.

**Significance Statement**

While rubella typically causes mild disease in children and adults and is preventable through vaccination, serious complications develop from congenital rubella syndrome, which is caused by infection during pregnancy and is a leading cause of vaccine-preventable birth defects worldwide. There are no specific antivirals that treat rubella, so we have addressed this by screening a drug repurposing library. Downstream validation studies in diverse and relevant cell types reveal two nucleoside analogs that have been previously tested in humans and are active against rubella virus. These are promising candidates for further development as antiviral treatments.

## Introduction

Rubella is a contagious disease caused by rubella virus, a member of the genus *Rubivirus* within the Matonaviridae family (1, 2). Humans are the only known reservoir of the rubella virus, and the virus is contracted through the respiratory route (3). After the virus establishes infection in the respiratory tract, the individual becomes viremic, and the virus infects diverse secondary sites, including the skin, which leads to a rash (1). For most individuals, rubella is ultimately cleared. However, during pregnancy, rubella infection can cause fetal death, stillbirth, or a constellation of severe birth defects, known as congenital rubella syndrome (CRS) (4). During congenital infection, the fetus is infected transplacentally during the maternal viremic phase and, depending on the stage of gestation, this can lead to multiple fetal defects, including the classic triad of cataracts, congenital heart defects, and sensorineural deafness (3).

The live-attenuated vaccine for rubella virus is protective, and its introduction into national immunization programs has resulted in the elimination of the virus from the United States in 2005 and many countries across the world thereafter (1). However, globally, there remains a large number of unvaccinated persons, leading to an estimated 30,000–50,000 CRS cases in 2020 (5). Elimination of rubella and CRS is a goal of the World Health Organization (4). As vaccine coverage for rubella improves and CRS is eliminated in developed countries, less common complications become more evident. For example, immunodeficiency-related vaccine-derived rubella viruses (iVDRVs) have been observed in nearly 100 patients with immunodeficiencies and several clinically immunocompetent adults (6, 7). These patients were originally identified with cutaneous granulomas; however, additional sites of infection such as lung, liver, and bone marrow have subsequently been observed (8). Sequencing has revealed prevalent amino acid substitutions and an increased number of substitutions over time, indicating a continuous sequence evolution of iVDRVs in these immunocompromised patients (9). Each iVDRV isolate is unique and has evolved to become less cytopathic and persistent, leading to severe morbidity in these patients (10).

There are no approved antivirals against the rubella virus, and there are few studies exploring the activity of existing drugs against this virus. Therefore, we set out to identify small molecules active against the rubella virus by screening an in-house repurposing library of ∼3,500 drugs that include FDA-approved drugs and bioactives with known targets, including antivirals. Among the candidates are two nucleoside analogs, NM107 and AT-527, which are active against the rubella virus. Both drugs were originally developed against the hepatitis C virus (HCV) (11). Since NM107 and AT-527 have been tested in humans and AT-527 has been shown to be safe and well tolerated, we suggest that these antivirals should be developed further for the treatment of rubella virus infections.

## Results

### High-throughput screen for antivirals against rubella virus

**Fig. 1.**
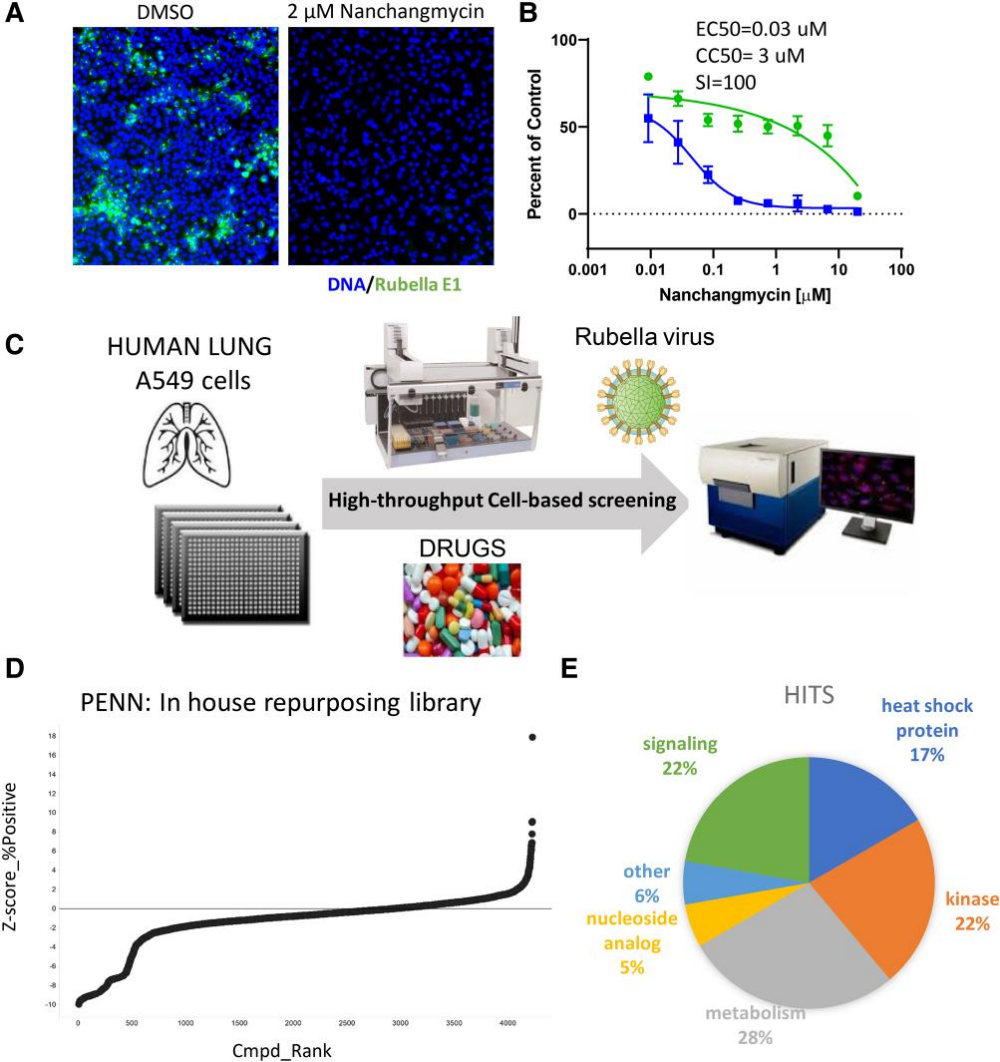
High-throughput screen identifies small molecules active against rubella virus. A) Human respiratory A549 cells were treated with vehicle (DMSO) or 2 μM nanchangmycin and infected with rubella RA27/3 virus (MOI = 0.2). At 48  hpi, cells were fixed and stained for viral infection (antirubella E1, green) and cell number (Hoechst 33342, blue). 10× magnification. B) A549 cells were treated with indicated concentrations of nanchangmycin and infected with rubella RA27/3 virus (MOI = 0.2). At 48 hpi, cells were fixed and stained (antirubella E1, Hoechst 33342). Automated microscopy and image analysis were used to quantify the cell number (green circles) and percent infection (blue squares) normalized to vehicle control (DMSO). Data are presented as mean ± SD (*n* = 3 independent biological replicates). C) Schematic overview of the high-throughput screening assay. D) Rank order of experimental drug activity by *Z*-score in the A549 rubella high-throughput screen. E) Pie chart of the 18 candidates identified from the primary screen annotating potential targets classes.

We developed a high-throughput, high-content, cell-based assay to monitor rubella virus infection of the human respiratory cell line A549, as the respiratory tract is infected in humans. Indeed, A549 cells have previously been shown to be permissive to rubella infection (12). We infected A549 cells with the rubella virus vaccine strain (RA27/3) and monitored infection using an antibody to the viral glycoprotein E1 (Fig. 1A) (13). We previously found that the broad-spectrum antiviral nanchangmycin can inhibit infection of a large number of diverse RNA viruses in several cell types, including A549 cells (14), and thus we tested the activity of nanchangmycin against rubella virus. To evaluate the utility of nanchangmycin as a positive control for our assay development, we performed a dose–response assay for nanchangmycin using automated fluorescent microscopy to quantify the cell number as a surrogate for cell viability and percent infection (Fig. 1A). We found that nanchangmycin inhibited rubella virus infection with a half-maximal inhibitory concentration (IC50) of 0.03 µM and a half-maximal cytotoxicity concentration (CC50) of 3 µM. The selectivity index (SI = CC50/IC50) of 100 (Fig. 1B) indicates that the antirubella efficacy of nanchangmycin is substantially higher than its cytotoxicity in A549, and thus, this drug can be used as a positive control in our screening assay.

**Fig. 2.**
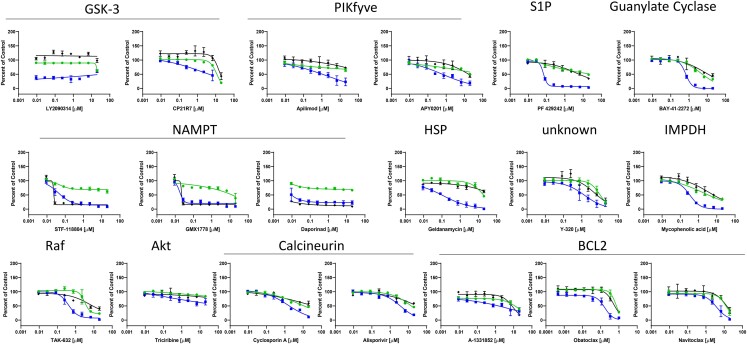
Validation of the candidate antiviral drugs in A549 cells. Dose–response analysis of the indicated inhibitors in A549 cells. Infection (blue squares), cell number (green circles), and ATPLite (black diamonds) are shown as Percent of Control. Data are presented as mean ± SD (*n* = 3 independent biological replicates). IC50, CC50, and SI for each drug are listed in Table S2.

A schematic of the experimental scheme for the high-throughput screen is shown in Fig. 1C, and optimization using nanchangmycin led to a *Z*′ of 0.55. We screened our in-house library of ∼3,500 drugs that include ∼1,500 FDA-approved drugs and another ∼2,000 drug-like molecules against defined molecular targets with validated pharmacological activity. The library contains 678 known kinase inhibitors, 435 annotated cancer therapeutics, 190 epigenetic regulators, 411 antiviral/infectives, and 596 G protein-coupled receptor and ion channel regulators. We previously screened this library against SARS-CoV-2 (15). We screened this repurposing library in A549 cells at 1 µM and quantified the percentage of infected cells and the cell number for each well (Fig. 1B and C). Using a relaxed cutoff of >60% inhibition and >60% cell viability compared with vehicle alone, we identified 18 candidates, and their target classes are shown in Fig. 1E, and Table S1. Among the candidates were three inhibitors of heat-shock proteins (geldanamycin, KW-2478, and NMS-E973), three nicotinamide phosphoribosyltransferase (NAMPT) inhibitors (STF-118804, GMX1778, and daporinad), two GSK-3 inhibitors (LY2090314 and CP21R7), along with additional signaling and metabolic regulators. We repurchased powders and validated the antiviral activity of these candidates in a dose–response assay in A549 cells (Fig. 2A and Table S2).

We found that the GSK-3 inhibitor LY2090314 was very active (SI > 2000), while another GSK-3 inhibitor CP21R7 was not (SI = 1). To determine whether LY2090314 is targeting GSK-3 or potentially a different target, we tested two additional GSK-3 inhibitors, AZD2858 and CHIR99021. We found that these compounds had little activity (Table S2), suggesting that LY2090314 may be antiviral through a GSK-3-independent mechanism. We also validated the PIKfyve inhibitor APY0201, known to block entry of viruses that use endosomal entry routes and found robust activity (SI = 102). We also tested a second PIKfyve inhibitor (apilimod) and found that this drug also has activity (SI > 8). All three of the NAMPT inhibitors identified in the screen (STF-118804, GMX1778, and daporinad) validated with high potency (SI > 500). Given that NAMPT impacts energetics in the cell, we used an independent ATPLite assay to monitor cytotoxicity, and we found that all three NAMPT inhibitors deplete ATP content in A549 cells with IC50s of 0.01 μM for STF-118804, 0.02 μM for GMX1778, and 0.01 μM for daporinad, suggesting that they are cytotoxic.

Of the additional candidates tested, we validated the S1P inhibitor PF-429242 (SI = 298), the guanylate cyclase inhibitor BAY-41-2272 (SI = 7), the heat-shock protein inhibitor geldanamycin (SI > 163), the inosine-5'-monophosphate dehydrogenase (IMPDH) inhibitor mycophenolic acid (SI = 6), and Y-320 that has an unknown target (SI = 7). In contrast, the MAPK signaling inhibitors TAK-632 and triciribine known to target Raf and Akt, respectively, showed little activity. The calcineurin inhibitor cyclosporin A identified in the screen and a second-generation inhibitor alisporivir also showed little activity. Lastly, we identified the BCL2 inhibitor A-1331852 in the screen and thus tested this inhibitor along with obatoclax and navitoclax, which also target BCL2. We found little activity against rubella in A549 cells with significant toxicities. Altogether, these data show that we could validate the activity of most targets, only 14 had an SI > 5.

### Host-targeted antivirals show broad activity

**Fig. 3.**
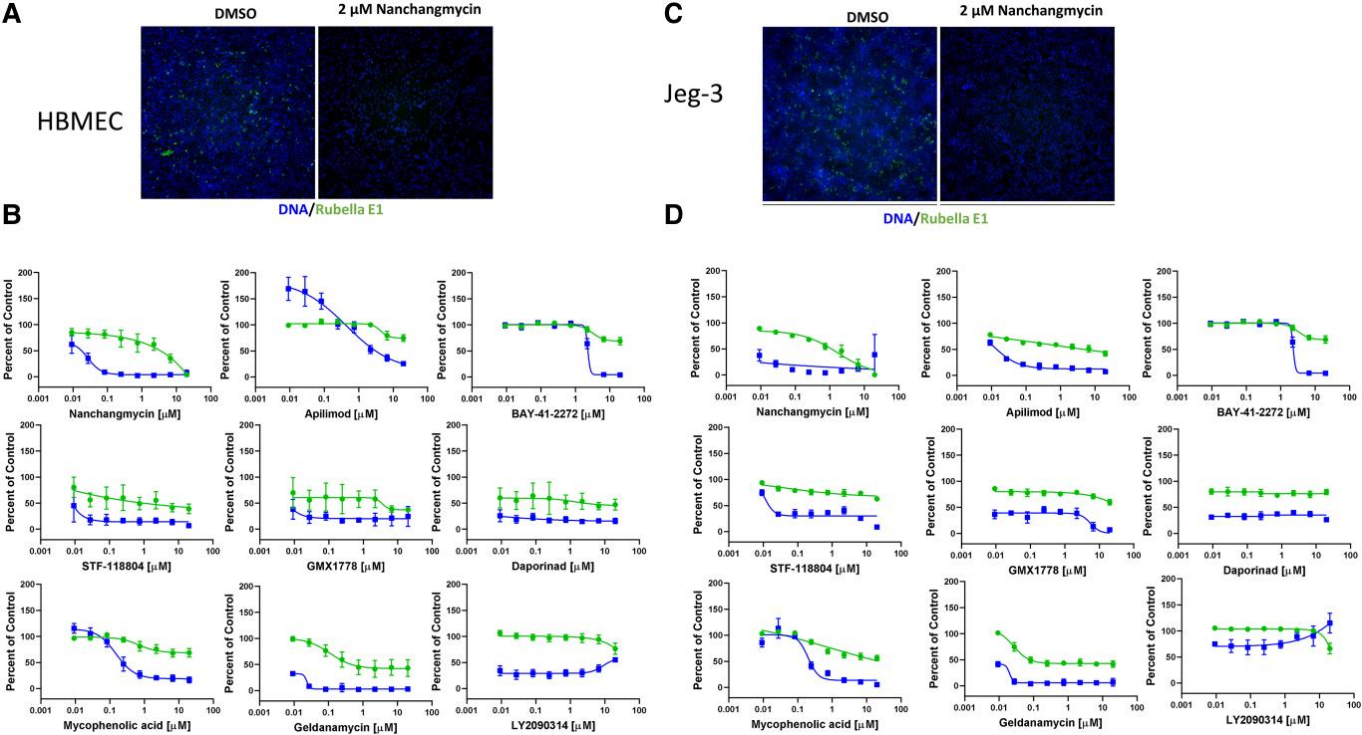
Host-targeted drugs are also active in additional cell types. A) HBMECs were treated with vehicle (DMSO) or 2 μM nanchangmycin and infected with rubella virus (MOI = 0.1). At 48 hpi cells were fixed and stained for viral infection (antirubella E1, green) and cell number (Hoechst 33342, blue). 10 × magnification. B) Dose–response analysis of the indicated inhibitors in HBMEC. Infection (blue squares) and cell number (green circles) are shown. Data are presented as mean ± SD (*n* = 3 independent biological replicates). IC50, CC50, and SI for each drug are listed in Table S2. C) Human trophoblast Jeg-3 cells were treated with vehicle (DMSO) or 2 μM nanchangmycin and infected with rubella virus (MOI = 0.05). At 48 hpi cells were fixed and stained for viral infection (antirubella E1, green) and cell number (Hoechst 33342, blue). 10 × magnification. D) Dose–response analysis of the indicated inhibitors in Jeg-3 cells. Infection (blue squares) and cell number (green circles) are shown. Data are presented as mean ± SD (*n* = 3 independent biological replicates). IC50, CC50, and SI for each drug are listed in Table S2.

Rubella virus can infect diverse tissues in vivo, and viruses can show tissue-specific dependencies on host factors. Therefore, we set out to determine if these host-directed drugs have activity against rubella virus in two additional cell types. First, we developed assays in human brain microvascular endothelial cells (HBMECs) as a model for endothelial cells. We again used nanchangmycin to optimize the assay (Fig. 3A and B). We then performed dose–response studies against the panel of drugs that were identified as active against rubella in A549 cells (Table S2). We found that most of the drugs with an SI > 5 in A549 cells also showed activity in HBMEC: the NAMPT inhibitors (STF-118804, GMX1778, and daporinad), the PIKfyve inhibitors (APY0201 and apilimod), the HSP90 inhibitor geldanamycin, the GSK-3 inhibitor LY2090314, the IMPDH inhibitor mycophenolic acid, and Y-320. In addition, the BCL2 inhibitor obatoclax showed activity in HBMEC against rubella.

Since rubella can cause congenital infections, we next developed assays in the human trophoblast cell line Jeg-3. We found that these cells are very permissive to rubella infection and again optimized the assay using nanchangmycin. We found that nanchangmycin was active against the rubella virus in these cells (Fig. 3C and D). Next, we performed dose–response studies against the panel of drugs identified as active against rubella in A549 cells and found that almost all of the drugs that were active in HBMEC were also active in Jeg-3 cells: the NAMPT inhibitors (STF-118804, GMX1778, and daporinad), the PIKfyve inhibitors (APY0201 and apilimod), the IMPDH inhibitor mycophenolic acid, and the BCL2 inhibitor obatoclax. In contrast, the GSK-3 inhibitor LY2090314 and Y-320 showed no activity and no toxicity in Jeg-3 cells. Lastly, the HSP90 inhibitor geldanamycin had high toxicity in the Jeg-3 precluding analysis. Altogether, these data suggest that many, but not all, of the host-directed drugs are antiviral against rubella virus infection across cell types.

### Nucleoside analog NM107 is antiviral against rubella virus

**Fig. 4.**
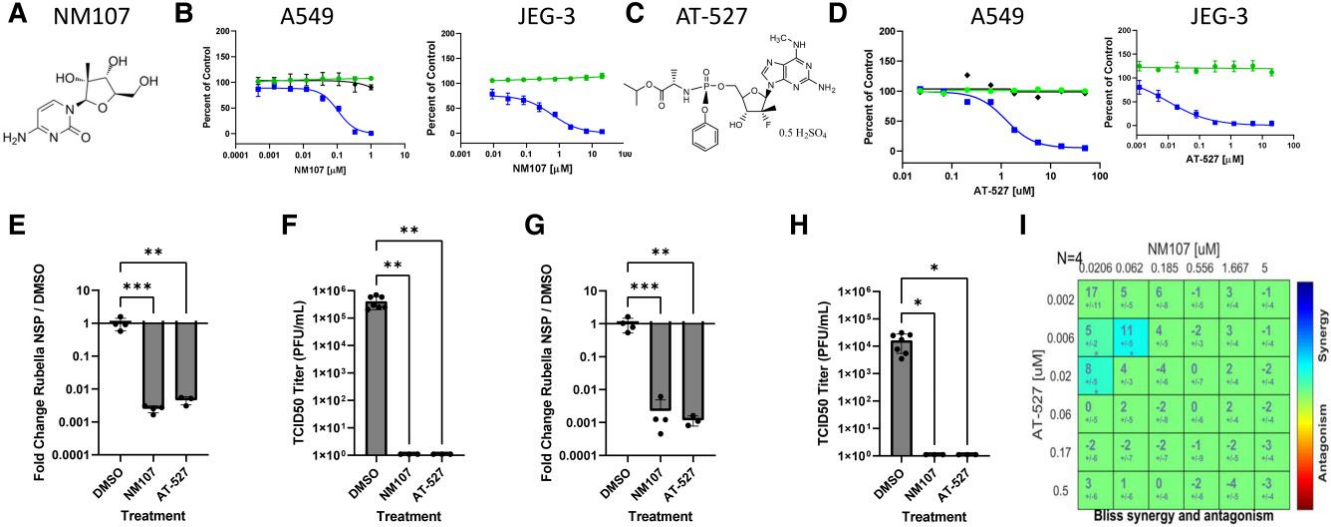
Nucleoside analogs NM107 and AT-527 are antiviral against rubella virus. A) Structure of NM107. B) Dose–response analysis of NM107 in A549 and Jeg-3 cells. Infection (blue squares), cell number (green circles), and ATPLite (black diamonds, A549 only) are shown. Data are presented as mean ± SD (*n* = 3 independent biological replicates). C) Structure of AT-527. D) Dose–response analysis of AT-527 in A549 and Jeg-3 cells. Infection (blue squares), cell number (green circles), and ATPLite (black diamonds, A549 only) are shown. Data are presented as mean ± SD (*n* = 3 independent biological replicates). E) A549 cells pretreated with vehicle or 10 μM of the indicated drugs and infected with rubella for 48 h, and total RNA was subject to qRT–PCR analysis of viral infection. Data are presented as mean ± SD for reduction compared with vehicle control (*n* = at least three independent biological replicates; **: *P* < 0.01, ***: *P* < 0.001, one-way ANOVA). F) Viral titers from supernatants from E. Data are presented as mean ± SD for reduction compared with vehicle control (*n* = at least three independent biological replicates) (**: *P* < 0.01, one-way ANOVA). G) Jeg-3 cells pretreated with vehicle or 10 μM of the indicated drugs and infected with rubella for 48 h, and total RNA was subject to qRT–PCR analysis of viral infection. Data are presented as mean ± SD for reduction compared with vehicle control (*n* = at least three independent biological replicates) (**: *P* < 0.01, ***: *P* < 0.001, one-way ANOVA). H). Viral titers from supernatants from G. Data are presented as mean ± SD for reduction compared with vehicle control (*n* = 3 independent biological replicates) (*: *P* < 0.05, one-way ANOVA). I). BLISS analysis of the 2 × 2 combination of NM107 and AT-527 in Jeg-3 cells showing additivity. Data are presented as mean values of excess over BLISS for *n* = 4 independent biological replicates. The statistical significance was determined by using a one-sample Student's t test (*P < 0.05).

The largest class of approved antivirals is nucleoside analogs, which can be incorporated by virally encoded polymerases into the newly elongating viral nucleic acids to inhibit infection. The repurposing library we screened contains ∼150 nucleoside analogs, many of which are known antivirals. In addition to the host-directed small molecules identified in the primary screen, we also identified a single nucleoside analog from the library, 2′-C-methylcytidine (NM107) (Fig. 4A). NM107 was originally developed as an inhibitor of the HCV NS5B RdRp (16). We performed dose–response studies and found that in A549 cells, NM107 has an IC50 of 20 nM and a CC50 of >20 µM, making the SI > 817 (Fig. 4B and Table S2). NM107 is more active against rubella than HCV in cell-based assays (17). In HBMEC, NM107 has an IC50 of <10 nM and a CC50 of >20 μM, making the SI > 2,000. In Jeg-3 cells, NM107 has an IC50 of 600 nM and a CC50 of >20 μM with a SI > 34. Therefore, NM107 is a potent antiviral against rubella virus across cell types.

### Nucleoside analog AT-527 is antiviral against rubella virus

While our bioactive screening library contains ∼150 nucleoside analogs, we also assembled a panel of 23 nucleoside analogs found to have antiviral activity against additional RNA viruses, including SARS-CoV-2 (18). We arrayed these in an 8-pt dose–response assay and included NM107 as a positive control. We screened this panel of nucleoside analogs against rubella virus in both A549 cells and Jeg-3 cells. In addition to NM107, we identified the double prodrug of 2′-fluoro-2′-C-methylguanosine (AT-527/bemnifosbuvir hemisulfate) as having antiviral activity against rubella virus (Fig. 4C and D). AT-527 is another nucleotide analog originally developed for the treatment of HCV (19). We found that in A549 cells, the IC50 is 2.25 µM with an SI > 9, and in Jeg-3 cells, the IC50 is 10 nM with an SI > 2,063 (Table S3). There are striking differences in the IC50s between cell types, which is likely due to differences in the processing of nucleoside analogs into active triphosphorylated forms.

We next performed orthogonal assays to determine the magnitude of the inhibition of rubella infection upon treatment with either NM107 or AT-527. We treated A549 cells with vehicle control, 10 µM NM107, or 10 µM AT-527 and observed >100-fold reduction in viral replication as measured by qRT–PCR (Fig. 4E). We also performed virus yield studies in A549 cells and found that NM107 and AT-527 reduced viral titers by ∼100,000-fold, at the limit of detection (Fig. 4F). We next treated trophoblast Jeg-3 cells with NM107 or AT-527 and monitored viral replication by qRT–PCR and found that both drugs led to >100-fold reductions in viral RNA (Fig. 4G). We also determined if these nucleoside analogs impacted virus production. We found that NM107 and AT-527 reduced viral titers by ∼10,000-fold, below the limit of detection (Fig. 4H).

RNA viruses can rapidly evolve resistance to monotherapy, and combinations can be synergistic. Since NM107 is a pyrimidine analog and AT-527 is a purine analog, we considered that the combination may be synergistic. Therefore, we performed 2 × 2 combination studies with these two nucleoside analogs in a 6-pt dose–response and used a BLISS model to determine whether these drugs interact (20). We found that the combination of NM107 with AT-527 is additive, potentially allowing them to be used in combination (Fig. 4I).

### Nucleoside biosynthesis inhibitors are antiviral

**Fig. 5.**
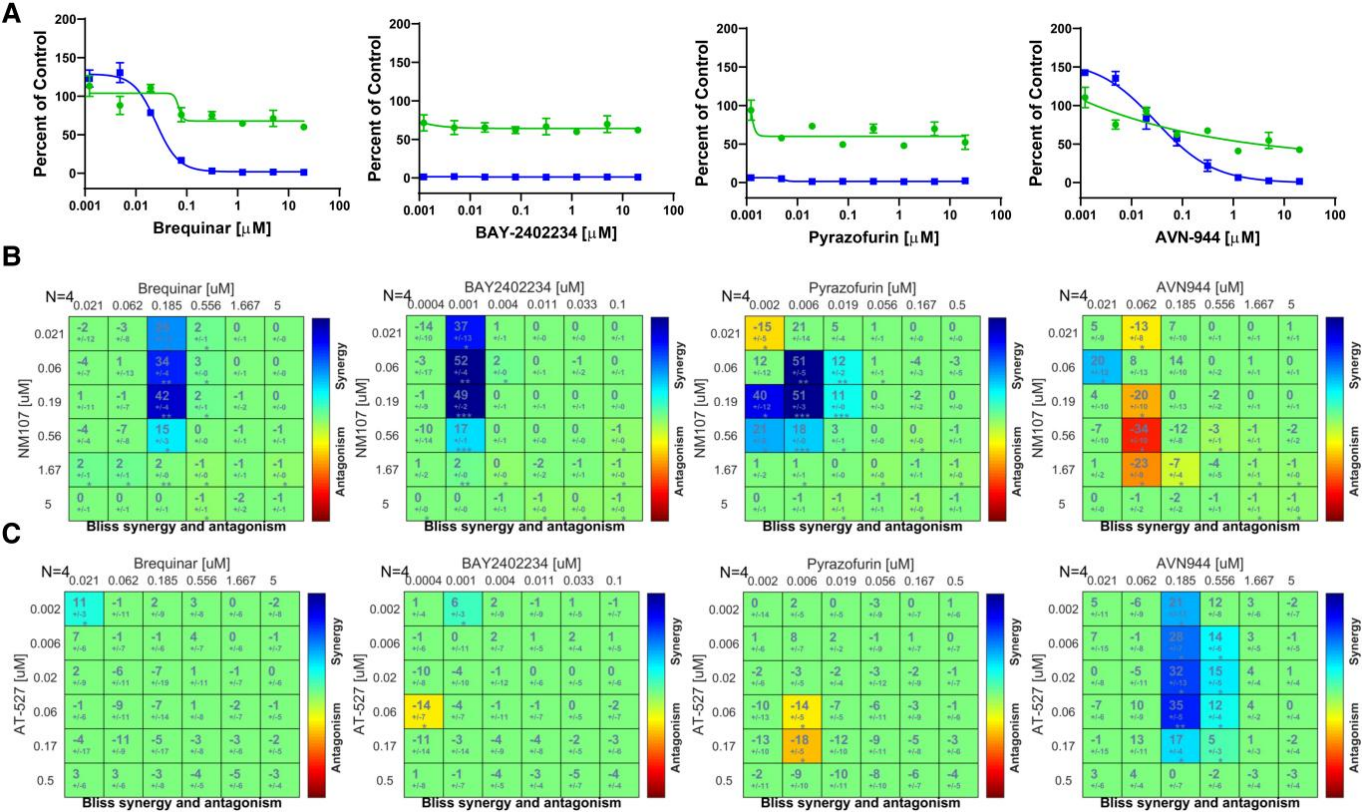
Combinations of nucleoside analogs with biosynthesis inhibitors show synergy. A) Dose–response analysis of the indicated nucleoside biosynthesis inhibitors in Jeg-3 cells. Infection (blue squares) and cell number (green circles) are shown. Data are presented as mean ± SD (*n* = 3 independent biological replicates). B) BLISS analysis of the 2 × 2 combination of NM107 with the indicated nucleoside biosynthesis inhibitors in Jeg-3 cells. Data are presented as mean values of excess over BLISS for *n* = 4 independent biological replicates. The statistical significance was determined by using a one-sample Student's t test (*P < 0.05; ** *P* < 0.001; *** *P* < 0.0001). C) BLISS analysis of the 2 × 2 combination of AT-527 with the indicated nucleoside biosynthesis inhibitors in Jeg-3 cells. Data are presented as mean values of excess over BLISS for *n* = 4 independent biological replicates. The statistical significance was determined by using a one-sample Student's t test (*P < 0.05; ** *P* < 0.001).

Diverse studies have found that nucleoside biosynthesis inhibitors can have broad antiviral activity, due to their dependence on high levels of nucleosides for RNA replication. Indeed, the IMPDH inhibitor mycophenolic acid was a validated candidate that emerged from the primary screen but showed higher toxicity in A549 cells than in Jeg-3 cells (Figs. 2 and 3, and Table S2). Previously, we found that a panel of nucleoside biosynthesis inhibitors, including AVN-944, a second-generation IMPDH inhibitor, was antiviral against SARS-CoV-2 (18). In addition to the purine biosynthesis inhibitor AVN-944 that inhibits guanosine biosynthesis, we also identified three pyrimidine biosynthesis inhibitors: brequinar and BAY-2402234 that target DHODH and pyrazofurin that targets the downstream enzyme UMPS as an antiviral against SARS-CoV-2. Moreover, we found that the combination of nucleoside biosynthesis inhibitors with nucleoside analogs showed synergistic antiviral activity against SARS-CoV-2 (18). Therefore, we tested the activity of these four nucleoside biosynthesis inhibitors during rubella infection of Jeg-3 cells (Fig. 5A). As expected, we found that all four drugs show some toxicity, but we also observed significant antiviral activity against rubella virus at concentrations that were nontoxic (Fig. 5A).

This allowed us to test combinations of each of the four nucleoside biosynthesis inhibitors with either the cytosine analog NM107 or the guanosine analog AT-527. We found that the combinations of the pyrimidine nucleoside analog NM107 with inhibitors of pyrimidine biosynthesis (brequinar, BAY-2402234, or pyrazofurin) showed synergy, while the combination with the purine biosynthesis inhibitor AVN-944 showed no synergy, with modest antagonism (Fig. 5B). In contrast, combinations of the guanosine analog AT-527 with the purine biosynthesis inhibitor AVN-944 showed synergy, while combinations with the pyrimidine biosynthesis inhibitors did not (Fig. 5C). These data suggest that decreasing concentrations of endogenous nucleosides can potentiate the activity of nucleoside analogs of the same class.

## Discussion

There are no approved antivirals to treat rubella infection and only a few reports describing therapeutic efforts to treat persistent rubella infection in a small case series (10, 12). The lack of a small animal model for rubella virus makes the development of antivirals against rubella virus even more challenging. While interferon treatment can have broad antiviral activities, it was found that this did not improve rubella virus antigen in skin lesions in a case study (10). In contrast, treatment with nitazoxanide reduced rubella antigen in a patient and showed antiviral activity in cultured cells infected with rubella virus. The mechanism of action of nitazoxanide is unknown but potentially promising. Given the dearth of antivirals, we screened a repurposing library for drugs with activity against rubella virus in a human respiratory epithelial cell line and found a number of host-targeted drugs with activity. We tested additional cell types, including a model of human trophoblasts, to determine those small molecules that have antiviral activity more broadly. We found that the natural product nanchangmycin is potently and broadly active, suggesting that we may consider this for future development. We also found that inhibitors of host biology, including NAMPT, PIKfyve, and IMPDH, showed activity. PIKfyve inhibitors likely block viral entry (21). NAMPT inhibitors are very active but have toxicity concerns given their role in cellular energetics. Another class of host-targeted agents includes the nucleoside biosynthesis inhibitors. Mycophenolic acid inhibits IMPDH, which is a rate-limiting step in guanosine biosynthesis. We tested a second-generation IMPDH inhibitor (AVN-944), as well as three pyrimidine biosynthesis inhibitors, and found that they all displayed antiviral activity in human trophoblast Jeg-3 cells.

In addition to the inhibitors of host biology, we also identified the nucleoside analog NM107 as a potent antiviral against rubella virus infection across diverse cell types. NM107 was under development for HCV infection (22). Due to its low bioavailability, the prodrug NM283 was developed and reached clinical trials, but was discontinued due to gastrointestinal toxicity (23, 24). We previously identified nucleoside analogs with antiviral activity against SARS-CoV-2, and we mined the literature for additional nucleoside analogs with published activity against SARS-CoV-2 (18). We tested this panel of nucleoside analogs in dose–response against rubella virus and identified a second nucleoside analog, AT-527, as having antiviral activity against rubella virus. AT-527 was also initially developed for HCV infection, showed in vivo activity in a clinical trial, and was well tolerated (19, 25). This suggests that AT-527 may have the potential for repurposing against rubella virus infection. Future studies will determine the mechanism by which these nucleosides block RNA replication. While we focused our studies on the vaccine strain of rubella virus, we suggest that these would be active against diverse strains of rubella virus, since the RNA polymerases are highly conserved. Our previous studies showed synergistic antiviral activity of nucleoside analogs with nucleoside biosynthesis inhibitors against SARS-CoV-2 (18), and thus, we tested these combinations in rubella virus. We found that combinations of nucleoside analogs with nucleoside biosynthesis inhibitors show synergy, suggesting that decreasing pools of nucleosides may generally boost the utilization of nucleoside analogs to augment their antiviral activity. Altogether, these studies suggest that repurposing nucleoside analogs may be a straightforward route to treating rubella infection and should be pursued in the future.

## Materials and methods

### Viruses and cells

A549 cells were obtained from ATCC (CCL-185) and cultured in DMEM, supplemented with 10% (v/v) fetal bovine serum (FBS; Sigma-Aldrich), 1% (v/v) penicillin/streptomycin (Invitrogen), and 1% (v/v) glutamax (Invitrogen). Jeg-3 cells were obtained from Dr Coyne (Duke University) and cultured in minimum essential medium (MEM) with Earle's salts supplemented with 10% (v/v) FBS and 1% (v/v) penicillin/streptomycin. HBMECs were obtained from Dr Coyne (Duke University) and cultured in RPMI-1640 (Corning), supplemented with 10% (v/v) FBS, 10% (v/v) Nu-serum (Corning), 1% (v/v) penicillin–streptomycin, 1× MEM nonessential amino acids (Invitrogen), 1 mM sodium pyruvate (Invitrogen), and 10 µg/mL endothelial cell growth supplement (Fisher Scientific). All cell lines were maintained at 37°C and 5% CO_2_. Rubella RA27/3 vaccine strain was obtained from the Centers for Disease Control and Prevention (CDC). Rubella RA27/3 was generated in Vero CCL81 cells (ATCC) from supernatants and viral titer was determined by TCID_50_ assay.

### High-throughput screen

A549 cells were plated in 384 well plates at 3,000 cells/well, HBMEC were plated at 7,000 cells/well, or Jeg-3 cells were plated at 3,000 cells/well in 20 μL growth media. The next day, 50 nL of drugs were added to assay wells, yielding a final concentration of 1 µM. The positive control nanchangmycin (*n* = 32) and the negative control DMSO (*n* = 32) were spotted on each assay plate. One hour later, cells were infected with rubella virus at a multiplicity of infection (MOI) = 0.234 for A549s, MOI = 0.1 for HBMEC, MOI = 0.05 for Jeg-3 cells). MOIs were optimized by titrating rubella virus stock in each cell line to achieve comparable infection rates across cell lines. HBMECs were spin-infected by centrifuging at 1,250*×g* for 1 h at 25°C. Cells were fixed at 48 h post infection (hpi) in 4% formaldehyde/phosphate-buffered saline (PBS) for 15 min at room temperature and then washed twice with PBS. Cells were blocked with blocking buffer [2% bovine serum albumin in PBS with 0.1% Triton X-100 (PBST)]) for 1 h and incubated with primary antibody (mouse antirubella E1, CDC, 1 μg/mL) overnight at 4°C. Cells were washed three times with PBST and incubated in secondary (1:1000 antimouse Alexa Fluor 488 and 5 μg/mL Hoechst 33342) for 1 h at room temperature. Cells were washed three times with PBST and imaged using ImageXpress Micro (Molecular Devices). Cells were imaged with a 10× objective capturing four sites per well. The total number of cells and the percentage of infected cells were measured using the MetaXpress 5.3.3 cell scoring module. The aggregated percent infection of the DMSO and 2 µM nanchangmycin control wells on each assay plate were used to calculate *Z*′-factors. Sample well infection was normalized to aggregated DMSO plate control wells and expressed as percentage of control (POC = [%Infection sample/Average %Infection DMSO] × 100) and *Z*-score (*Z* = [%Infection sample − Average %Infection DMSO]/Standard Deviation %Infection DMSO) in Spotfire (PerkinElmer). Candidate hits were selected as compounds with POC <40% and viability >60%, compared with vehicle control.

### Dose–responses

Drugs were purchased as powders from Selleckchem, MedchemExpress, and MedKoo and suspended in DMSO. Drugs were arrayed in 8-pt dose–response in 384 well plates. Infections were performed using the screening conditions. DMSO (*n* = 32) and 2 µM nanchangmycin (*n* = 16) were included on each validation plate as controls for normalization. Infection at each drug concentration was normalized to aggregated DMSO plate control wells and expressed as POC (POC = %Infection sample/Average %Infection DMSO cont). A nonlinear regression curve fit analysis (GraphPad Prism 8) was performed on POC infection and cell viability using log10 transformed concentration values to calculate IC50 values for infection and CC50 values for cell viability. SI was calculated as a ratio of the drug's CC50 and IC50 values (SI = CC50/IC50).

### qRT–PCR

A549 cells were plated in 6-well plates at 5 × 10^5^ cells/well, HBMEC were plated at 6 × 10^5^ cells/well, or Jeg-3 cells were plated at 5 × 10^5^ cells/well. Twenty-four hours later, vehicle or drugs were added at the indicated concentrations. One hour later, cells were infected with rubella virus (MOI = 0.17 for A549s, MOI = 0.07 for HBMEC, MOI = 0.05 for Jeg-3 cells) and HBMEC were spin-infected by centrifuging at 1,250*×g* for 1 h at 25°C. MOIs were optimized by titrating rubella virus stock in each cell line to achieve comparable infection rates across cell lines. At 48 hpi, total RNA was purified using Trizol (Invitrogen) followed by the RNA Clean and Concentrate kit (Zymo Research). cDNA synthesis was performed with random hexamers and Moloney murine leukemia virus reverse transcriptase (Invitrogen). Gene-specific primers to rubella RA27/3 NSP and GAPDH were used (Table S4). SYBR green qPCR master mix (Applied Biosystems) amplified targets using the QuantStudio 6 Flex qRT–PCR system (Applied Biosystems). Relative quantities of viral and cellular RNA were calculated using a relative standard curve method where quantification was performed with respect to a 5-point standard curve generated from serial dilutions of a pooled reference generated by combining each sample cDNA for the experiment. Viral RNA was normalized to GAPDH RNA.

### Viral titers

A549 or Jeg-3 cells were plated, treated, and infected as for the qRT–PCR experiments. At 4 hpi, cells were washed twice with DPBS (Invitrogen) to remove any nonadsorbed virus and 2 mL of appropriate culture medium treated with vehicle or drug was added. At 48 hpi, cell supernatants were collected and titered by TCID_50_ assay.

### TCID_50_ assay

A549 cells were plated in 96 well plates at 25,000 cells/well and incubated overnight before inoculating with two-fold serial dilutions of viral stock or virus-containing supernatant to determine viral titer. At 24 hpi, plates were processed for microscopy and imaged as above. Titers were calculated using the Reed–Muench method (26).

### Quantification and statistical analysis

*P*-values for qRT–PCR experiments were obtained by performing one-way ANOVA, assuming equal SD, with multiple comparisons using Dunnett's correction for multiple tests on relative copy number values from at least three independent experiments. For TCID_50_ experiments, the same analysis was performed on titers from at least three independent experiments. For multiple comparisons, each condition was compared with vehicle control (DMSO). Visualization of data was performed using GraphPad Prism 9. The statistical parameters for experiments are described in the figure legends. *n* indicates the number of independent experiments performed, and significance is defined as *P* < 0.05.

## Supplementary Material

pgad256_Supplementary_Data

## Data Availability

All data are included in the manuscript and/or supporting information.
