# Supplementary material for: Nucleoside analogs NM107 and AT-527 are antiviral against rubella virus
Source: PNAS Nexus. 2023 Aug 3;2(9):pgad256. doi: 10.1093/pnasnexus/pgad256 (PMC10479830; doi:10.1093/pnasnexus/pgad256)
Supplement: pgad256_Supplementary_Data [file pgad256_supplementary_data.pdf]

PNAS nexus

Supplementary Information for
Nucleoside analogs NM107 and AT-527 are antiviral against rubella virus

Mark Dittmar[1,3], Kanupriya Whig[2], Jesse Miller[1], Brinda Kamalia[2], Suganthi Suppiah[4], Ludmila Perelygina[4], Kathleen E. Sullivan[5], David C. Schultz[2], Sara Cherry[1,2,3]

Sara Cherry
University of Pennsylvania 472A Stemmler Hall
3450 Hamilton Walk
Philadelphia, PA 19104
Email: cherrys@pennmedicine.upenn.edu

This PDF file includes:

Tables S1 to S4
SI References

Table S1. Primary screen hits and target classes

Product Name	Target	POC_TotalCells	Zscore_TotalCells	POC_%Positive	Zscore_%Positive
A-1331852	BCL2	77.30	-5.50	24.78	-7.32
Apilimod	PIKfyve	73.09	-6.86	24.57	-7.49
APO866	NAMPT	67.77	-5.87	17.31	-8.28
BAY 41-2272	guanylate cyclase (GC	76.05	-3.24	15.78	-6.74
CP21R7	Wnt/beta-catenin	98.37	-0.40	38.49	-5.99
Cyclosporin A	calcineurin	62.70	-6.79	33.07	-6.70
Geldanamycin	HSP90	85.77	-2.59	15.71	-8.44
GMX1778	NAMPT	71.34	-3.88	16.77	-6.66
KW-2478	HSP90	80.39	-3.57	6.13	-9.40
LY2090314	GSK-3	89.57	-2.75	34.91	-5.28
Mycophenolic acid	other	63.11	-6.71	30.77	-6.93
NM107	HCV	100.11	0.03	4.16	-9.51
NMS-E973	HSP90	74.06	-4.72	10.43	-8.97
PF429242	S1p	66.18	-8.63	7.49	-9.18
STF-118804	NAMPT	70.25	-7.94	21.95	-8.33
TAK-632	Raf	79.99	-3.64	30.57	-6.95
Triciribine	Akt	82.56	-4.05	38.06	-5.85
Y-320	unknown	81.56	-4.92	20.67	-8.46

Table S2. Candidate molecules dose-response

			A549				HBMEC				JEG3			A549	A549 S
**Product Name**	**target**	**units**	**IC50**	**CC50**	**SI**	**ATPLite**	**IC50**	**CC50**	**SI**	**ATPLite**	**IC50**	**CC50**	**SI**	primary	Validat
**A-1331852**	BCL2	uM	6.13	>20	>3	9.136	0.4889	3.389	7	0.4102	13.47	18	1	yes	no
**apilimod**	PIKfyve	uM	2.46	>20	>8	>20	0.4819	>20	>42	>20	0.01127	>20	>1775	yes	yes
**BAY-41-2272**	sGC	uM	0.6619	4.693	7	8.753	2.613	>20	>8	>20	2.219	>20	>9	yes	yes
**CP21R7**	GSK3	uM	>20	11.4	1	15.5	>20	>20	1	>20	>20	>20	1	yes	no
**Cyclosporin A**	cyclophilin	uM	1.487	8.7	6	>20	18.59	>20	1	>20	9.012	12.28	1	yes	yes
**Daporinad**	NAMPT	uM	0.01	>20	>2000	0.01	0.01	>20	>2000	0.01	<0.009145	>20	>2187	yes	yes
**Geldanamycin**	HSP90	uM	0.12	>20	>163	>20	<0.01	0.1139	11	>20	<0.009145	0.02645	2	yes	yes
**GMX1778**	NAMPT	uM	0.02	20	1120	0.02215	<0.01	>20	>2000	<0.01	<0.009145	>20	>2187	yes	yes
**LY2090314**	GSK3	uM	0.01	>20	>2000	>20	0.01	>20	>2000	>20	>20	>20	1	yes	yes
**Mycophenolic acid**	IMPDH	uM	0.3324	1.928	6	3.936	0.11	>20	>182	>20	0.1996	>20	>100	yes	yes
**NM107**	HCV NS5B	uM	0.02	>20	>817	>1	<0.01	>20	>2000	>20	0.5925	>20	>34	yes	yes
**PF-429242**	S1P	uM	0.06702	20.00	298	>20	>20	>20	1	>20	>20	>20	1	yes	yes
**STF-118804**	NAMPT	uM	0.04	>20	>500	0.01	<0.01	>20	>2000	<0.01	0.01095	>20	>1826	yes	yes
**TAK-632**	Raf	uM	0.4	3.1	8	>20	4.495	>20	>4	>20	5.562	>20	>4	yes	yes
**Triciribine**	Akt	uM	>20	>20	1	>20	>20	>20	1	>20	>20	>20	1	yes	no
**Y-320**	unknown	uM	1.431	9.98	7	5.222	1.66	>20	>12	>20	>20	1.05	1	yes	yes
**nanchangmycin**	unknown	uM	0.03	3.0	98	8.835	0.02377	4.785	>201	3.333	<0.009145	1.694	>185	yes	yes
**APY0201**	PIKfyve	uM	0.20	20.0	102	17.09	0.3361	12.1	36	17.06	0.01294	5.987	463	related	yes
**AZD2858**	GSK3	uM	>20	>20	1	>20	>20	>20	1	>20	>20	>20	1	related	no
**CHIR99021**	GSK3	uM	11.06	>20	>2	>20	6.965	>20	3	>20	>20	>20	1	related	no
**navitoclax**	BCL2	uM	3.84	13.3	3	13.29	1.768	4.812	3	4.355	9.514	11.15	1	related	no
**obatoclax**	BCL2	uM	0.1675	0.6967	4	0.5344	0.0405	>1	>25	>1	0.1106	>20	>181	related	no

Table S3. Nucleoside analog screen dose response

Product Name	A549_RUBELLA_IC50	A549_RUBELLA_CC50	A549_RUBELLA_SI	JEG3_RUBELLA_IC50	JEG3_RUBELLA_CC50	JEG3_RUBELLA_SI
6-mercaptopurine	8.83	> 20	> 2	2.17	18.77	9
6-thio-dG	> 20	> 20	1	0.12	0.59	5
6-thioinosine	9.5	> 20	> 2	1.96	> 20	> 10
abacavir	> 20	> 20	1	> 20	> 20	1
AT-527	2.25	> 20	> 9	0.01	> 20	> 2063
azacytidine	0.51	3.37	7	56.35	13.29	1
azaguanine	> 20	> 20	1	> 20	> 20	1
azathioprine	15.63	> 20	1	6.76	> 20	> 2
CF-1743	> 20	> 20	1	> 20	> 20	1
EIDD-1931	8.56	> 20	> 2	> 20	> 20	1
favipiravir	> 100	> 100	1	> 100	> 100	1
Galidesivir hydrochloride	> 20	> 20	1	> 20	> 20	1
GS-441524	> 20	> 20	1	> 20	> 20	1
maribavir	> 20	> 20	1	> 20	> 20	1
mercaptopurine	9.31	> 20	> 2	1.85	20	11
Molnupiravir	> 20	> 20	1	> 20	> 20	1
NM107	0.001	> 20	> 20000	0.03	> 20	> 604
remdesivir	> 20	> 20	1	6.21	4.85	1
riboprine	7.2	> 20	> 2	13.63	> 20	1
thiamiprine	> 20	> 20	1	1.05	8.38	8
thioguanine	5.41	> 20	> 3	0.29	0.8	3
tubercidin	0.07	0.04	1	0.95	0.16	1
Valnivudine	> 20	> 20	1	> 20	> 20	1

Table S4. Gene-specific RT-qPCR primers

Primer name	Sequence (5' -> 3')	Source
Rubella RA27/3 NSP Forward	CCGGTGGGCCTTATAACTTAAC	This paper
Rubella RA27/3 NSP Reverse	GAAACAACATCGCGCACTTC	This paper
GAPDH Forward	ACCAAATCCGTTGACTCCGACCTT	K. Rausch *et al*.
GAPDH Reverse	TCGACAGTCAGCCGCATCTTCTTT	K. Rausch *et al*.

SI References

1. K. Rausch *et al.*, Screening Bioactives Reveals Nanchangmycin as a Broad Spectrum Antiviral Active against Zika Virus. *Cell Rep* **18**, 804-815 (2017).
